# EGFR and HER2 expression in cervical cancer patients in Ibadan, Nigeria

**DOI:** 10.3332/ecancer.2023.1607

**Published:** 2023-10-02

**Authors:** Ajibike Ayomide Orekoya, Abbas A Abdus-Salam, Abdurasaq R Oyesegun, Atara I Ntekim, Ayorinde M Folasire, Clement A Okolo, Adeniyi A Olabumuyi, Adedamola A Dada, Adeniyi A Adenipekun

**Affiliations:** 1 Federal Medical Centre Ebute-Metta, Lagos 101211, Lagos State, Nigeria; 2 Radiation Oncology Department, University College Hospital, Ibadan 200212, Oyo State, Nigeria; 3 National Hospital, Abuja, Nigeria; 4 Pathology Department, University College Hospital, Ibadan 200212, Oyo State, Nigeria

**Keywords:** EGFR, epidermal growth factor receptor 1, HER1, HER2, human epidermal growth factor receptor 2, cervical cancer, overall survival, Nigeria

## Abstract

Cervical cancer is a leading cause of cancer-related deaths in developing countries, including Nigeria where it is the second most common female malignancy. Studies from elsewhere have demonstrated the relationship between epidermal growth factor receptor (EGFR) and human epidermal growth factor receptor 2 (HER2) and advanced cervical cancer. However, we are not aware of such studies in Nigerian patients. The main objective of the study was to determine the prevalence of EGFR or HER1 and HER2 protein expression in cervical cancers and to determine their impact on overall survival. Clinical data and formalin-embedded tissue blocks of 124 patients who presented in the Radiation Oncology Department, University College Hospital (UCH), from 2006 to 2015 and had their histological diagnosis at the Pathology Department, UCH were retrieved and analysed for EGFR and HER2 expression using immunohistochemistry. EGFR expression was analysed using the immunoreactivity score by Remmele and Stegner. HER2 was analysed using the Hercep® test kit guidelines. Survival analysis was done using Kaplan–Meier and Cox regression analysis. Missing data were reported as missing, not documented. EGFR (immunoreactivity score > 4) was overexpressed in 26.6% of the 124 cervical tissue samples tested. Most patients whose samples were positive for EGFR were young, had squamous cell carcinoma and advanced diseases. HER2 was overexpressed in two samples (1.6%). The 5-year overall survival rate of the patients was 28.3%. The 5-year survival rate of patients who were EGFR positive was 9.5% and 34.1% for those who were EGFR negative. Screening for EGFR should be considered in cervical cancer patients. HER2 was overexpressed in two cervical tissue samples in this study and may be of poor interest as a potential target in the management of cervical cancer patients. Large prospective multi-institutional studies should be considered to further explore the relationship between EGFR and survival in cervical cancer patients.

## Introduction

Cervical cancer is on the increase in developing countries but its incidence and mortality are reducing in developed countries where there is widespread screening, human papilloma virus (HPV) vaccination and prompt and adequate treatment [1]. The treatment of cervical cancer depends on the stage at presentation as well as other factors. However, the mortality is high in locally advanced and metastatic disease. There are many studies trying to investigate molecular biological markers which may have a role to play in the survival, prognosis and management of cervical cancer patients [2–10]. Studies on epidermal growth factor receptor 1 (EGFR or HER1) and human epidermal growth factor receptor 2 (HER2 neu) have been investigated in Caucasian and Asian patients with cervical cancer [7–26]. However, none of these proteins have been studied in indigenous Africans.

EGFR is a 170-kDa transmembrane glycoprotein receptor encoded by the HER-1 proto-oncogene located on chromosome 7p12. It is also known as HER1. EGFR is a transmembrane protein that is a receptor for the epidermal growth factor (EGF) family of extracellular protein ligands. EGFR is activated by binding with its specific ligands EGF and transforming growth factor-alpha (TGFα). After it is activated by its growth factor ligands, it is transformed from an inactive monomer form to an active homodimer. EGFR may also form heterodimers with other members of the ErbB receptor family which include ErbB2 (HER2/neu), ErbB3 (HER3) and ErbB4 (HER4). The dimerisation of EGFR stimulates its intrinsic protein kinase activity which results in the autophosphorylation of several tyrosine residues in the C domain of EGFR. This elicits downstream activation and signal transduction proteins leading to DNA synthesis, cell growth, differentiation and proliferation. EGFR is expressed in many normal tissues as well as many solid tumours like cervical cancer, lung adenocarcinomas, epithelial tumours of the head and neck and glioblastomas. In normal cervical tissues EGFR is expressed in the cytoplasm and basement membrane of cells but during cell differentiation there is a shift to the cytoplasm. The precise mechanisms by which EGFR dysregulation leads to oncogenicity are still under investigation as they are not well known. However, several processes have been implicated including the roles of coexpression of receptor ligands (EGF, TGFα and amphiregulin), EGFR gene amplification, reduced phosphatase levels, heterodimerisation and crosstalk with downstream and other members of the ErbB receptor family, and interactions with downstream and other cell signal transduction pathways, and viral proteins. In cervical cancer development, HPV infection is associated with cytoplasmic EGFR which is involved in cervical cancer aetiology. HPV’s genome encodes small proteins E6, E7 and E5 that mimic cancer-causing oncogenes. HPV type 16 E5 protein can activate EGFR by binding to the ATPase subunit of EGFR resulting in decreased degradation of EGFR receptors, increased EGFR recycling and EGFR overexpression. HPV E6 expression is also associated with increased EGFR expression. The increased growth rate of cervical cancer cell lines may be caused by the decreased stability of EGFR at the post-transcriptional level due to changes in functional levels of HPV E6/E7 proteins. EGFR mutations are rare in high-grade cervical lesions and invasive cervical cancer. This suggests that high-risk HPV proteins affect EGFR at the cellular level leading to overexpression and not genomic levels leading to mutations.

Monoclonal antibodies against EGFR include cetuximab and panitumumab. Other monoclonals in clinical development include zalutumumab, nimotuzumab and matuzumab. Small molecule tyrosine kinase inhibitors with anti-EGFR activity include geftinib, erlotinib, brigatinib and lapatinib.

HER2 is a transmembrane receptor protein that has a cysteine-rich extracellular ligand-binding domain, a hydrophobic membrane-spanning region and an intracellular tyrosine kinase domain. HER2 functions as a preferred partner for heterodimerisation with other members of the EGF receptor family (i.e., HER1 or ErbB1, HER3 and HER4). HER2 protein overexpression, gene amplification and mutation have been identified in a variety of cancer types which include breast cancer, gastrointestinal cancers like gastric, colorectal cancers, pancreatic cancer, oesophageal, biliary tract cancers lung cancer, salivary gland tumours, gynaecological cancers like ovarian, cervical, endometrial cancers and vulvar Paget’s disease, bladder and head and neck squamous cell carcinoma [16, 22, 27–30].

Before the introduction of the HERCEP test, 8%–77% of cervical carcinomas were seen to overexpress HER2 as evaluated by diverse methods [22, 29]. Some of these studies showed that HER2 overexpression in cervical cancer was associated with a worse prognosis [29]. Since the introduction of HERCEP test guidelines, HER2 expression in cervical cancer ranged from 0% to 4% [22, 16, 29–31].

HER2 overexpression has been associated with resistance to chemotherapeutic agents and is predictive of a favourable response to other agents [27].

Trastuzumab, an anti-HER2 antibody is an effective targeted therapy with significant efficacy in treating HER2-positive breast and gastric cancers (metastatic gastric and gastroesophageal cancers). In recent years the family of approved anti-HER2 agents has been expanding with the addition of small molecule inhibitors (e.g., lapatinib (reversible inhibitor of HER1 and HER2), neratinib, afatinib, canertinib (all irreversible inhibitors of HER1, HER2 and HER4)), antibodies (e.g., pertuzumab) and an antibody-drug conjugate (adotrastuzumab emtansine, T-DM1) [20, 27, 30, 32]. These agents have been used alone or in combination with other targeting agents or chemotherapy and have shown remarkably improved outcomes in patients with HER2-positive breast cancer [20, 27, 30, 32]. Inhibitors of downstream targets of HER2 include everolimus (mTOR), BKM120 (PI3K/AKT), BEZ-235 (PI3K/AKT/ mTOR), GS-1101 (PI3K), NVP-BKM120 (PI3K), GDC-0941 (PI3K), GSK458 (PI3K/mTOR), GDC-0980 (PI3K/mTOR) and PI-103 (PI3K/mTOR). Hsp90 inhibitors include tanespimycin, retaspimycin and AUY922 [27]. An anti-HER2 vaccine is being investigated [27].

The aim of this study was to determine if EGFR and HER2 proteins were overexpressed in cervical cancer tissue samples and their relation to the survival of indigenous African cervical cancer patients.

## Materials and methods

### Patients

For this study, all patients who presented to the Radiotherapy Department of the University College Hospital (UCH), Ibadan, Oyo State, Nigeria, from January 2006 to December 2015 and who also had their pathological diagnosis done at the Pathology Department of UCH, Ibadan, Oyo State, Nigeria, were selected. Patients were only included in the analysis if their case notes and paraffin-embedded tissue samples were seen and if there was enough tumour tissue available for analysis.

### Inclusion criteria

All patients who were histologically diagnosed with cervical cancer in the Pathology Department also presented in the Radiation Oncology Department of UCH, Ibadan, Oyo, Nigeria, from 2006 to 2015.

### Exclusion criteria

Patients who had no histological diagnosis.

Patients who had missing case files.

Patients with scanty treatment records.

### Institutional review board approval

Ethical approval was sought and gotten from the Joint Ethical Committee of Ibadan/UCH, Ibadan.

### Immunohistochemistry protocol

The EGFR primary antibody used was Santa Cruz Biotechnology Inc. EGFR (A-10): sc-373746. The HER2 primary antibody used was Biocare c-erbB-2/HER2 antibody. The secondary antibody used was Elabscience E-IR-R217, 2-step plus poly horse radish protein anti-mouse/rabbit IgG detection system (with diaminobenzidene (DAB) solution) [33, 34].

Four micron tissue sections were cut from paraffin-embedded tissue blocks, picked up onto 3-aminopropyltriethoxysilane) slides and heat fixed on a hotplate for 2 hours at 60°C. Sections were dewaxed using xylene, cleared in ethanol and rehydrated in descending grades of concentration of ethanol. They were then transferred to distilled water for wash-off. Slides were washed with Trizma phosphate buffer saline (PBS) for 20 minutes. Heat-induced epitope retrieval was done in a water bath at 95°C–100°C in which slides were heated for 5 minutes at 120°C in a citrate buffer and left to cool there for 20 minutes. Endogenous peroxidase was blocked by incubating in hydrogen peroxidase for 10 minutes. Non-specific background staining was blocked by treatment with protein block. Excess protein block was then drained from the slides. Primary antibodies EGFR1 and HER2 were then applied and slides were incubated for 15 minutes at room temperature. Slides were washed with Trizma PBS for 20 minutes a second time. Primary antibody link (Match 3 pro) was applied and slides were incubated at room temperature for 15 minutes. Slides were washed with Trizma PBS for 20 minutes a third time. Horseradish peroxidase polymer was applied and slides were incubated at room temperature for 15 minutes. Slides were washed with Trizma PBS for 20 minutes a fourth time. DAB chromogen was applied for 5 minutes. The slides were rinsed in running tap water. Slides were counterstained with haematoxylin for 10 seconds. Slides were rinsed in running tap water a second time. The slides were then dehydrated and mounted with Dibutylphthalate polystyrene xylene (DPX) and cover slipped.

### Evaluation of staining

EGFR/HER1 expression was analysed using the immunoreactivity score by Remmele and Stegner score [35–37]. In the assessment of EGFR staining, the percentage of immunoreactive tumour cells was rated as follows: no staining = 0; less than 10% positive cells = 1; 10%–50% positive cells = 2; 51%–80% positive cells = 3; greater than 80% positive cells = 4 and the intensity of staining as follows: no colour reaction = 0, weak/mild colour reaction (1+); moderate (2+) and strong/intense colour reaction (3+). The grade of score expression was obtained by multiplying the percentage of immunoreactive tumour cells with intensity of staining. The final immunoreactivity scores were as follows: 0 = negative; 1–4 = weak; 5–8 = moderate; 9–12 = strong [15, 38, 39]. For analysis, the scores were grouped into two, those with weak scores, i.e., 0–4 were grouped as negative and those with moderate or strong scores, i.e., 5–12 were grouped as positive. As no score of 5 could be gotten by the multiplication of the percentage and intensity scores, final immunoreactivity scores ≥6 were positive samples.

HER2 was analysed using the Hercep® test kit guidelines for breast cancer [40]. A score of 0 was given for no staining or staining in less than 10% of the tumour cells. A score of 1+ for faint or barely perceptible membrane staining in greater than 10% of tumour cells or when cells exhibited incomplete membrane staining. A score of 2+ for moderate staining of the complete cell membrane in more than 10% of tumour cells. A score of 3+ was given for intense staining of the complete cell membrane in more than 10% of the tumour cells. HER2 overexpression was assessed as negative for scores of 1+ or 0 and positive for 3+ scores. A score of 2+ was considered indeterminate but could not be re-evaluated by fluorescence in situ hybridisation (FISH) as it was not available in UCH (the study centre).

### Survival analysis

Survival time was determined from case notes, calling the patients and their relatives to find out their survival status for those who defaulted follow-up or were referred to other centres. Patients who could not be reached were censored as alive at the last time they were seen in the hospital.

### Statistical analysis

Statistical analysis was performed using SPSSv25 for Windows software (SPSS Inc.) (formerly Statistical Package for the Social Sciences, now Statistical Product and Service Solution). Univariate analysis was done using chi-square statistics. Overall survival was defined as the time from diagnosis to death of any cause or last follow-up visit alive. Survival rates were assessed using survival curves according to Kaplan–Meier estimates while Mantel-Cox log rank tests were used to evaluate the differences. Differences in overall survival according to clinicopathologic characteristics and protein expression were analysed using Cox regression analysis. *p-*value > 0.10 were excluded stepwise in multivariate analysis, in the final step any factor with a *p-*value < 0.05 was included. The level of significance was determined as *p* < 0.05. Missing data were reported as unknown, not documented or missing. 

## Results

### Patient and tumour characteristics

A total of 124 patient case records met the selection criteria and their corresponding tissue samples were retrieved and analysed.

Clinicopathologic characteristics of patients included in this study are summarised in Table 1. The median follow-up time was 8.5 months (range 0–163 months) for all patients. About 23% of patients did not have any form of treatment.

### Clinicopathologic factors in relation to staining of EGFR, HER2

**Figure 1.**
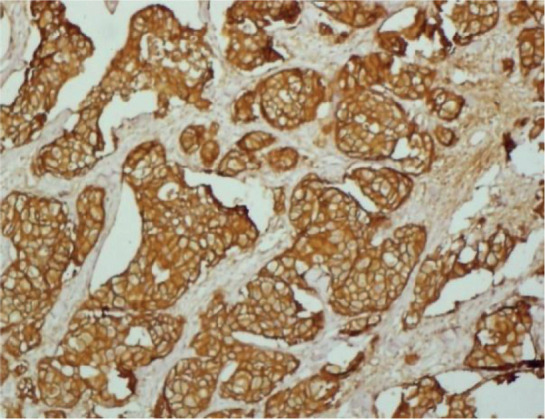
Strong EGFR staining in a cervical adenocarcinoma tissue sample.

**Figure 2.**
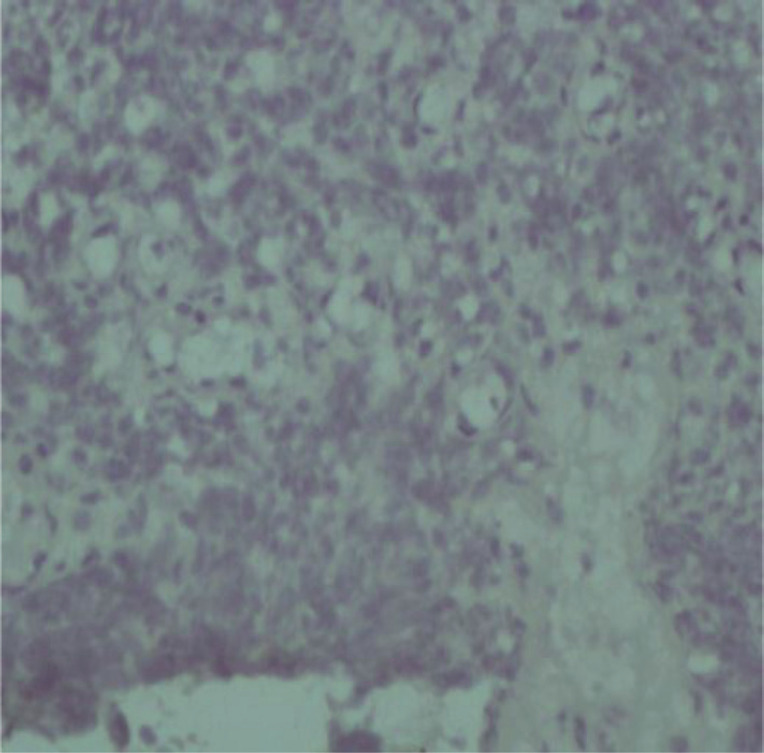
Negative EGFR staining in a cervical squamous cell carcinoma tissue sample.

**Figure 3.**
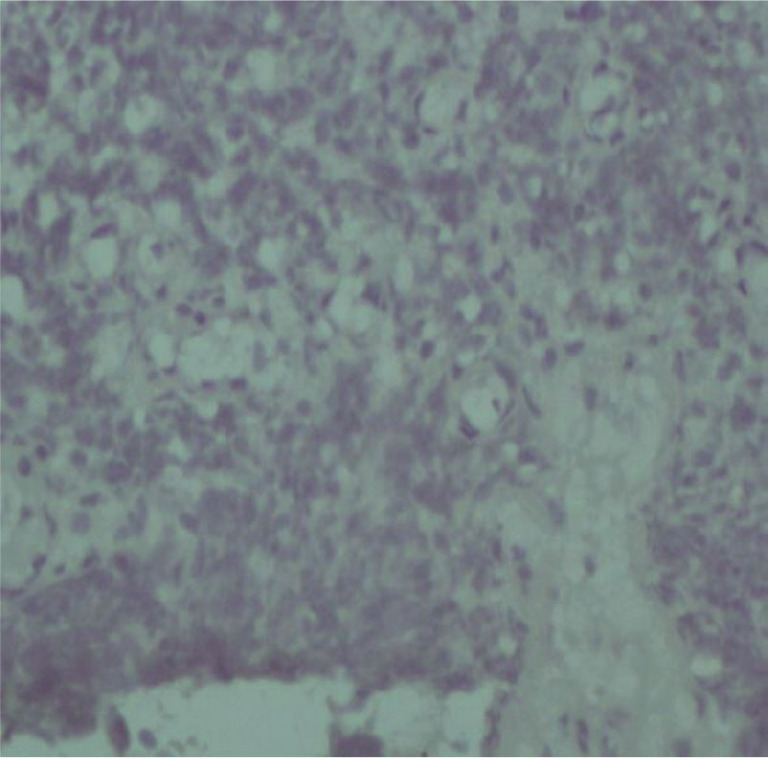
Negative HER2 staining in a cervical cancer tissue sample.

**Figure 4.**
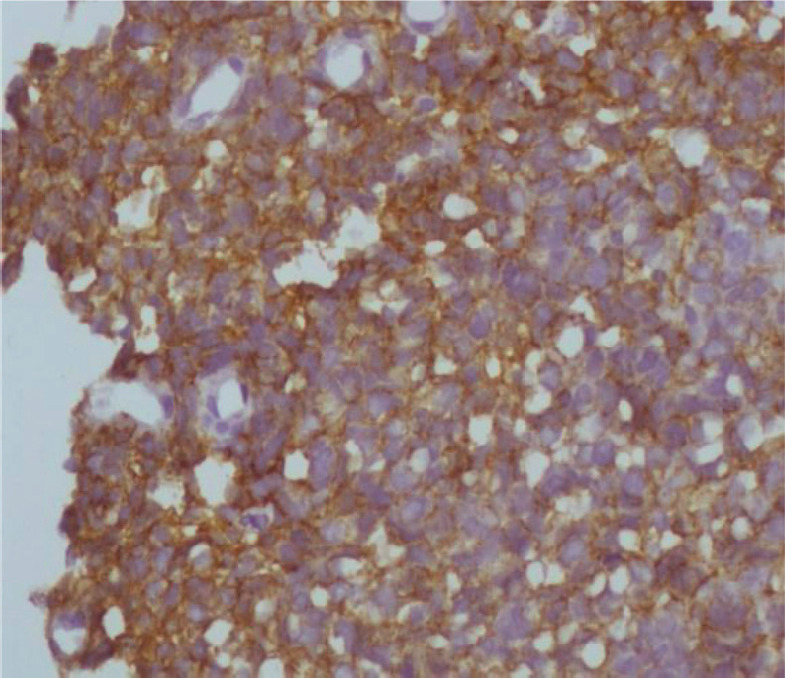
3+ positive HER2 staining in a cervical cancer tissue sample.

Immunohistochemistry was performed for EGFR and HER2. Figures 1–4 show representative positive and negative tumours for each protein. Healthy normal skin was used as a positive control for EGFR and HER2-positive breast cancer was used as a positive control for HER2.

Positive EGFR staining was present in 33/124 (26.6%) and positive HER2 staining was present in 2/124 (1.6%) of patients. No more statistical analysis was done with regard to HER2. However, EGFR was also overexpressed in the two patients who overexpressed HER2.

Photomicrographs of strong EGFR staining, negative EGFR staining, negative HER2 staining and 3+ HER2 staining are shown below in Figures 1–4, respectively.

There were no statistically significant relationships between EGFR and various clinicopathologic parameters as seen in Table 2.

### Positive EGFR staining is related to poor prognosis

**Figure 5.**
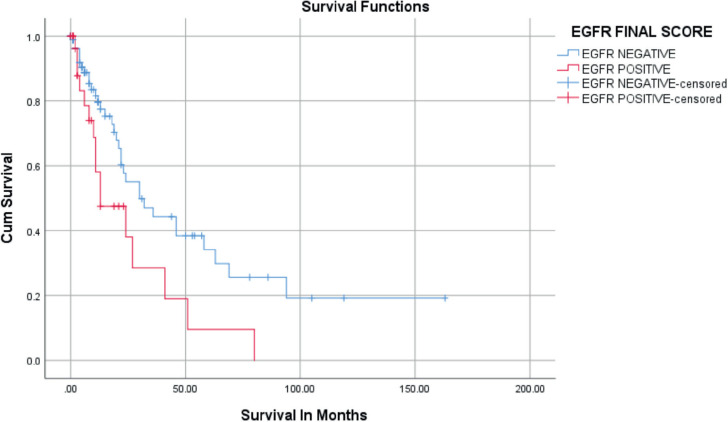
Kaplan–Meier survival curves for the relation of EGFR immunostaining with overall survival.

Positive immunostaining of EGFR was related to overall survival in univariate and multivariate analysis as seen in Table 3 and Figure 5. During the follow-up period, 49/124 (39.5%) patients died. The rest (75/124) were either alive or censored at variable lengths of time from the date of first diagnosis to the day they were last seen alive in the hospital. The overall survival at 2 years was 50.8%, standard error of 6.4. The overall survival at 5 years was 28.3%. The median survival of the patients was 27 ± 4.2 months (95% confidence interval, 18.7–35.3 months). Patients who had EGFR overexpression had a median survival time of 13 months which was statistically significantly shorter than those who were EGFR negative (30 months) (Figure 5). HER2 was overexpressed in only two patients (1.6%), so no survival analysis was done. On Cox regression analysis for overall survival, including age, stage and histological type and negative EGFR staining was independently related to better overall survival (hazard ratio = 0.233; 95% CI = 0.055–0.990; *p* = 0.048) (Table 3).

## Discussion

Our study is one of the first to emerge from Nigeria and the results appear to be in tandem with the findings of similar works from other institutions.

Of the 124 cervical tissue samples analysed, EGFR was overexpressed (using the immunoreactivity score described by Remmele and Stegner ≥ 6) in 33 patients (26.6%). Several studies have shown that EGFR was overexpressed in 6%–100% of cervical cancer tissue samples [6, 7, 12, 16, 17, 18, 23, 31, 38, 41–49]. This large range in the overexpression of EGFR cervical cancer tissue samples may be due to differences in the study’s methodology (scoring systems used, techniques used, detection methods, lack of standardised assays, assay cutoff points and the type of EGFR clones used). Many of the earlier studies used either quantitative means (percentage of cells stained) or qualitative means (intensity of staining). In this study, the Remmele and Stegner scoring system was used which adopts both quantitative and qualitative scores which are multiplied together to give a final immunoreactivity score. The lack of a standardised assay for determining EGFR status is particularly problematic. A universal method to evaluate tumour EGFR status should be adopted.

Some large studies showed an association of EGFR overexpression with younger age [31], histological type (more frequently and strongly overexpressed in squamous cell carcinomas than adenocarcinomas and adenosquamous carcinomas) [16, 44–46, 50], increasing tumour size, lymph node metastases and recurrence [6, 31, 43, 44, 48, 51]. However, EGFR overexpression was not found to be associated with known prognostic factors like age, stage and histology in this study. This is similar to some other studies which had small sample sizes and heterogeneous groups of patients in stage and histology [49].

Several studies have investigated the intensity of EGFR expression in different histologies. EGFR has been found to stain more strongly in squamous cell carcinomas than adenocarcinomas and adenosquamous carcinomas [16, 44–46, 50, 52]. The average percentage of squamous cell carcinoma overexpressing EGFR is 51% as compared with 38% for adenosquamous carcinomas and 23% for adenocarcinomas [6]. In this study, 87.9% of EGFR-positive samples were squamous cell carcinomas and 6.1% each were adenocarcinomas and adenosquamous carcinomas. EGFR was not overexpressed in adenoid cystic carcinoma and sarcoma samples. This pattern of EGFR overexpression is similar to findings by Skomedal et al [18], Lindström et al [50], Hale et al [45]. This differing expression of EGFR in different tumour histologies may indicate different impacts on carcinogenesis in different cancer types. However, the relationship between EGFR overexpression and histological subtypes did not attain statistical significance.

Skomedal *et al* [18] showed that advanced stages were associated with EGFR overexpression [18]. A similar result was seen in this study where 93.8% of patients who overexpressed EGFR were late cases. However, this association was also not statistically significant. Larger prospective studies may further explore and confirm this relationship.

Giordano *et al* [53] saw that EGFR overexpression was more common in younger women although it was not significant. This is similar to findings in this study where EGFR was more commonly overexpressed in younger women (27.0%) than older women (26%) but the association was not statistically significant. The mean age of patients who overexpressed EGFR was younger than those who were EGFR negative.

In this study, patients who had EGFR overexpression had a median survival time shorter than those who were EGFR negative. It was statistically significant (*p =* 0.020). This is similar to other studies which showed a statistically significant association between EGFR overexpression and poorer survival [23, 42, 43, 45, 51, 54]. Other studies have reported an absence of a relationship between EGFR overexpression and outcome in cervical cancer [12, 16, 17, 18, 46, 47, 50]. These discrepancies in relation to EGFR as a predictor of outcome in cervical cancer patients may be due to the variety of techniques and scoring systems used, lack of standardised assays and the different reagents used as well as the sample size and heterogeneity of the samples. The association with poorer survival in this study may be due to EGFR overexpression leading to a more malignant phenotype with an aggressive biologic behaviour. It has been suggested that EGFR overexpression confers enhanced radioresistance and lower sensitivity to Cisplatin therapy in tumour cells [23]. *In vitro* and *in vivo* studies have demonstrated that EGFR overexpression is associated with greater resistance to radiation therapy and lower sensitivity to cisplatin in several tumour types [1, 12, 37, 39, 49]. Although the majority of patients who overexpressed EGFR had late disease at presentation on Cox regression analysis, EGFR overexpression was found to be an independent factor predisposing to poorer survival. Patients who overexpressed EGFR in this study might have had a better survival if they had been offered anti-EGFR monoclonal antibodies like cetuximab. Larger prospective studies may better investigate the relationship between EGFR and survival in cervical cancer patients in Nigeria. The data presented in this study are supportive that EGFR status could be used to identify cervical cancer patients who may be eligible for anti-EGFR therapies.

HER2 was overexpressed in two cervical tissue samples (1.6%) in this study. This is at variance with earlier studies in which non-standardised scoring systems were used in which HER2 overexpression was seen in 38.7%–94% [17, 23, 55–57]. However, with the adoption of a more universally accepted HERCEP test scoring system, the value dropped to 0%–3.9% [16, 29–31] which is similar to what was seen in this study as the Hercep test guidelines was used. The original aim of this study was to find the prevalence of HER2 expression in cervical cancer samples and its correlation with other clinical variables. However, as only two samples (1.6%) were HER2 positive no further analysis could be done. Therefore, HER2 appears to be of poor interest as a potential target in the treatment of cervical cancer.

Synchronous expression of EGFR and HER2 has been found to be associated with lower survival rates [6, 16, 23]. This has been attributed to an increase in intrinsic tumour aggressiveness produced by the presence of homodimerisation or heterodimerisation, which confers enhanced radioresistance and lower sensitivity to cisplatin therapy to tumour cells. Heterodimer formation between EGFR and HER2 favours the recruitment of a greater number of intracellular signals involved in growth, survival and metastasising capacity of tumour cells [23]. EGFR and HER2 heterodimers are more potent than EGFR homodimers because HER2 produces broader and longer activation of cellular growth and proliferation signals. In other tumours, EGFR and HER2 co-expression has been associated with an increase in tumour aggressiveness and a worse prognosis, in comparison with either receptor expression [23]. In this study, the relationship between the synchronous expression of EGFR and HER2 and survival could not be assessed as HER2 was expressed in only two of the tissue samples.

The strengths of this study lie in the fact that it is the first of its kind in Nigerian cervical cancer patients and it has contributed to the knowledge of EGFR and HER2 expression as well as their relationship to survival in cervical cancer patients. This study could also serve as a template for further research (larger prospective studies and clinical trials) and in the generation of hypotheses. However, there are several limitations of this study which include the lack of a standard method for evaluating EGFR expression which interpreted results, and comparison with other studies difficult. Other limitations include its being a retrospective study. The majority of patients were lost to follow-up, a number of case notes were lost and tissue samples were destroyed/not found, so could not be used for the study and it was a single institutional study limiting the ability to translate these findings to other settings compared with prospective multi-institutional studies.

## Limitations of the study

The study was limited by its retrospective nature in which a lot of case notes used did not contain complete data. Also, the majority of the patients were lost to follow-up and could not reached by phone call. Also, a lot of case notes were missing. Cervical tissue samples for the majority of the patients to be used for the study were not found and of those found a lot could not be used for the study. Lack of funds made the possibility of visiting patients to determine their living status difficult. It was a relatively small study.

However, despite all these limitations, this study will contribute to the knowledge of the management of cervical cancer patients. It could serve as the template for further research (larger prospective studies) and in the generation of hypothesis.

## Conclusion

In conclusion, EGFR was overexpressed in 26.6% of the 124 patients whose cervical tissue blocks were found and its overexpression was significantly associated with poorer survival. However, the majority of those who overexpressed EGFR presented with late-stage disease, squamous cell carcinoma were younger, which may play a part in the poorer survival in this cohort of patients. HER2 was overexpressed in two cervical tissue samples in this study and may be of poor interest as a potential target in the management of cervical cancer patients. It appears that those who overexpressed during screening for EGFR may be considered for more aggressive treatment as the median survival of EGFR-positive patients was worse than EGFR-negative patients. As the cost of the test is a challenge to our patients, the National Health Insurance Authority, government, non-governmental Organisations and teaching hospitals should subsidise the cost of having the test done. A universal method to evaluate EGFR status in tumours should be adopted. Larger prospective multi-institutional studies may be required to validate the findings in this study.

## Conflicts of interest

The authors declare no conflicts of interest.

## Funding

Nil.

## Figures and Tables

**Table 1.** Patient and tumour factors.

Age at diagnosis Median Range	*n* – 124 56 years 28–83 years	
FIGO stage IA IB IIA IIB IIIA IIIB IVA IVB Missing	*N* 1 8 8 18 23 29 20 15 2 (details not recorded)	% 0.8 6.6 6.6 14.8 18.9 23.8 16.4 12.3 1.6
Histology Squamous cell carcinoma Adenocarcinoma Adenosquamous carcinoma Adenoid cystic carcinoma Sarcoma	109 9 3 2 1	87.9 7.3 2.4 1.6 0.8

**Table 2.** EGFR expression association with some variables.

	EGFR positive	EGFR negative	*p*-value
	33 (26.6%)	91 (73.4%)	
Age < 60 years	20 (60.6%)	54 (59.3%)	0.899
Stage ≥ IIB	30 (93.8%)	75 (83.3%)	0.117
Non squamous cell carcinoma	4 (12.1%)	11 (12.1%)	0.966

**Table 3.** Unadjusted and adjusted hazard ratios of variables using Cox regression analysis.

Variable	Unadjusted			Adjusted		
	Hazard ratio	95% CI	Sig	Hazard ratio	95% CI	Sig.
Age < 60 years	0.581	0.322–1.024	0.060	0.579	0.146–2.292	0.436
Squamous cell carcinoma	0.288	0.122–0.684	0.005	0.024	0.003–0.208	0.001
Stage < IIB	0.357	0.150–0.846	0.019	0.703	0.170–2.92	0.628
EGFR negative	0.498	0.271–0.912	0.024	0.233	0.055–0.990	0.048
